# *PDF1.5* Enhances Adaptation to Low Nitrogen Levels and Cadmium Stress

**DOI:** 10.3390/ijms221910455

**Published:** 2021-09-28

**Authors:** Zhimin Wu, Dong Liu, Ningyan Yue, Haixing Song, Jinsong Luo, Zhenhua Zhang

**Affiliations:** 1 Southern Regional Collaborative Innovation Centre for Grain and Oil Crops in China, College of Resources and Environmental Sciences, Hunan Agricultural University, Changsha 410128, China; wuzhimin@caas.cn (Z.W.); liudongzh@163.com (D.L.); yue493790266@163.com (N.Y.); shx723@163.com (H.S.); 2 Institute of Bast Fiber Crops, Chinese Academy of Agricultural Sciences, Changsha 410221, China; 3 National Centre of Oilseed Crops Improvement, Hunan Branch, Changsha 410128, China

**Keywords:** abiotic stress, cadmium adaptation, low nitrogen, environment adaptation ability, nutrient element distribution, plant defensin

## Abstract

Environmental acclimation ability plays a key role in plant growth, although the mechanism remains unclear. Here, we determined the involvement of *Arabidopsis thaliana* PLANT DEFENSIN 1 gene *AtPDF1.5* in the adaptation to low nitrogen (LN) levels and cadmium (Cd) stress. Histochemical analysis revealed that *AtPDF1.5* was mainly expressed in the nodes and carpopodium and was significantly induced in plants exposed to LN conditions and Cd stress. Subcellular localization analysis revealed that AtPDF1.5 was cell wall- and cytoplasm-localized. *AtPDF1.5* overexpression significantly enhanced adaptation to LN and Cd stress and enhanced the distribution of metallic elements. The functional disruption of *AtPDF1.5* reduced adaptations to LN and Cd stress and impaired metal distribution. Under LN conditions, the nitrate transporter *AtNRT1.5* expression was upregulated. Nitrate transporter *AtNRT1.8* expression was downregulated when *AtPDF1.5* was overexpressed, resulting in enhanced transport of NO_3_^−^ to shoots. In response to Cd treatment, *AtPDF1.5* regulated the expression of metal transporter genes *AtHMP07*, *AtNRAMP4*, *AtNRAMP1*, and *AtHIPP3*, resulting in higher Cd accumulation in the shoots. We conclude that *AtPDF1.5* is involved in the processing or transmission of signal substances and plays an important role in the remediation of Cd pollution and LN adaptation.

## 1. Introduction

Environmental acclimation ability plays a crucial role in plant growth, and nutrient element transport is a key factor in environmental acclimation [1,2,3]. It has previously been shown that plants have a higher nitrogen assimilation efficiency when larger amounts of NO_3_^−^ are transported to shoots [4]. Shoots are the main sites of photosynthesis and metabolism in plants [5,6], and the translocation of NO_3_^−^ from roots to shoots has been reported to be one of the main processes contributing to plant growth and higher nitrogen-use efficiency [7]. With respect to NO_3_^−^ transport, nitrate transporter 1.5 (NRT1.5) is involved in xylem NO_3_^−^ loading [8], whereas nitrate transporter 1.8 (NRT1.8) functions in xylem NO_3_^−^ unloading [9]. Accordingly, the NRT1.5 and NRT1.8 genes play important roles in NO_3_^−^ distribution [10,11].

Metallic elements play vital roles in plant growth and stress resistance [12,13], with different metals having specific functions [14]. In plants, potassium (K) can promote the activation of enzymes and enhance photosynthesis, sugar metabolism, and protein synthesis, thereby enhancing the ability of plants to resist drought, cold, salt, alkaline stresses, diseases, and pests [15,16]. Calcium (Ca) is mainly found in plant cell walls, where it functions in stabilizing membrane structure and serves as a secondary messenger in signal transduction [17]. Manganese (Mn) is an essential factor in chlorophyll biosynthesis and functions as an activator of enzymes such as transphosphatase (hexokinase), dehydrogenase (α-ketoglutarate dehydrogenase), nitrate reductase, and dipeptidase [18]. Iron (Fe) is absorbed mainly in the form of Fe^2+^ chelate and functions as a component of enzymes and electron transporters [19,20]. Copper (Cu) functions as a component of plastids and as an electron transporter in the photosynthetic chain, thereby contributing to electron transfer and photophosphorylation. Cu is also a component of enzymes such as cytochrome oxidase, phenoloxidase, ascorbic acid oxidase, polyamine oxidase, and peroxide dismutase involved in respiratory metabolism [21,22].

Concerning metal transporters, it has been reported that more than 30% of proteins have metallic ion constituents, which play key roles as structural components or catalytic factors [23,24]. Among the proteins known to mediate metal transport are natural resistance-associated macrophage protein-1 (*NRAMP1*), *NRAMP4*, and heavy metal-associated isoprenylated plant protein-3 (*HIPP3*). *NRAMP4* is located in the vacuolar membrane and transports metals from the vacuole to the cytoplasm via a proton symporter [25,26]. *NRAMP1* is present in the plasma membrane [27] and has been shown to play a role in Fe uptake under Fe deficiency conditions [28]. *HIPP3* is a nuclear-localized zinc-binding protein that is a member of the detoxification superfamily and is involved in heavy metal transport [29,30,31]. It is necessary to explore its function for acclimation or defense in environmental change.

Plant defensin (PDF) genes are a class of small molecular proteins, each comprising 45 to 54 amino acids, and at least 13 genes have been identified in *Arabidopsis thaliana*. PDF genes primarily play roles in fungal resistance and Zn tolerance [32,33,34,35] and can be divided into two families, namely, PDF1 and PDF2. Among the PDF1 family genes, *PDF1.2*, which can be induced by jasmonate and ethylene, has been reported to play a role in pathogen resistance [35]. In the PDF2 family genes, *PDF2.1* has been reported to show tissue-specific expression and affects ammonium metabolism by regulating glutamine synthetase activity [36,37]. *PDF2.5* has been reported to mediate Cd accumulation and tolerance by promoting cytoplasmic Cd efflux via chelation, thereby enhancing Cd detoxification and apoplastic accumulation. *PDF2.6* functions in the chelation of Cd in *A. thaliana* [38,39]. The PDF family may play a key role in environmental acclimation improvement. We speculated that *PDF1.5* acts as a signal molecule to regulate the expression levels of nitrate and cadmium-related transporters and regulate the adaptability of plants to low nitrogen and cadmium stress.

We have previously elucidated the mechanisms whereby LN enhances nitrogen-use efficiency and characterized plant responses to Cd stress [39,40]. Ethylene (ET) and jasmonic acid (JA) are two major plant stress hormones, also known as stress hormones [41]. Their synthesis is induced by stress, which regulates a series of stress responses in plants. Previous studies have shown that *NRT1.5* and *NRT1.8*-mediated nitrate redistribution under stress conditions is regulated by ethylene/jasmonic acid (ET/JA) signals [8]. However, we were unable to identify any common mechanisms with respect to plant responses to environmental adaptation, especially for LN levels and the presence of Cd. Previous results showed that the expression of *AtPDF1.5* was involved with LN and Cd stress (Figure S1). In addition, as a PDF1 member, the function of *AtPDF1.5* is still unclear. Therefore, in the present study, we aimed to elucidate the function of the *AtPDF1.5* gene, from the *AtPDF1* family, in the adaptation to LN levels and Cd stress.

## 2. Results

### 2.1. AtPDF1.5 Enhances Adaptation to LN Levels and Cd Stress

**Figure 1:**
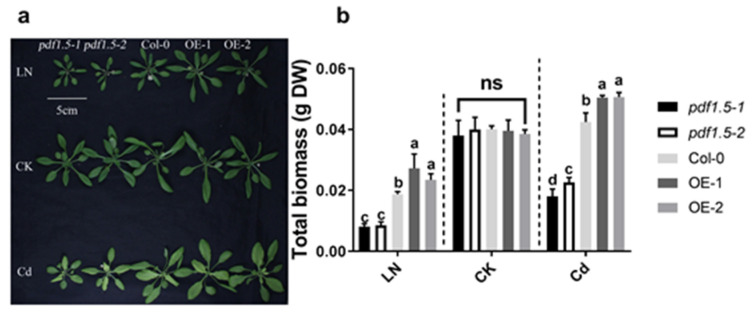
The phenotype for materials grown in low nitrogen (LN), control treatment (CK), or cadmium (Cd) conditions. (**a**) Phenotype for different materials—materials were harvested from 3-week-old hydroponically grown plants in either LN or Cd or CK conditions. (**b**) Total biomass for different materials. Bar height represents mean and error bars indicate standard deviation. Different letters on bars indicate significant differences based on Tukey’s HSD (*p* < 0.05). Six replicates were used for measurements.

Relative to the CK treatment, we found that the mutant *pdf1.5–1* and loss of function material *pdf1.5–2* had lower total biomass while the overexpression lines (OE-1 and OE-2) had higher total biomass in the LN and Cd treatments (Figure 1a,b). When Col-0 plants were grown under LN conditions, the expression of *AtPDF1.5* was upregulated in both shoots and roots (Figure S1a,c), whereas in plants treated with Cd, *AtPDF1.5* was highly upregulated in roots, but not significantly in shoots (Figure S1b,d).

### 2.2. AtPDF1.5 Influences Nitrogen Uptake and Translocation under LN Conditions

**Figure 2:**
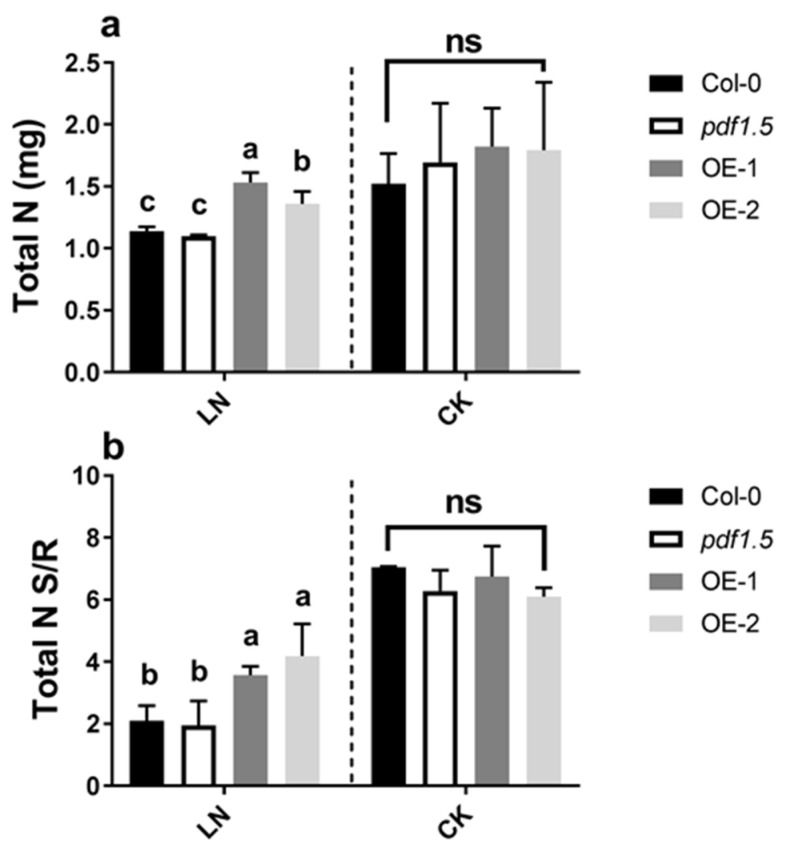
The involvement of *AtPDF1.5* in the nitrogen uptake and distribution in low nitrogen (LN) conditions. (**a**) Total nitrogen (N). (**b**) Total nitrogen (N) shoot/root. Six replicates were used for measurements. Bar height represents mean and error bars indicate standard deviation. Different letters on bars indicate significant differences based on Tukey’s HSD (*p* < 0.05). The statistical analysis was performed on different treatment genotypes. Six replicates were used for measurements.

Material verification for *AtPDF1.5* is shown in Figure S2. In the LN treatment, wild-type Col-0 and *pdf1.5* mutant seedlings showed no significant differences with respect to total nitrogen and shoot/root total nitrogen ratio (Figure 2a,b). Furthermore, relative to the wild-type Col-0, the *AtPDF1.5* overexpressing plants (OE-1 and OE-2) had a higher total N and shoot/root total nitrogen ratio (Figure 2a,b). Under the CK conditions, relative to the wild-type Col-0 seedlings, we detected no significant differences in total biomass (Figure 1a), total nitrogen (Figure 2a), or the shoot/root total nitrogen ratio (Figure 2b), in either *pdf1.5* or OE-1 and OE-2.

**Figure 3:**
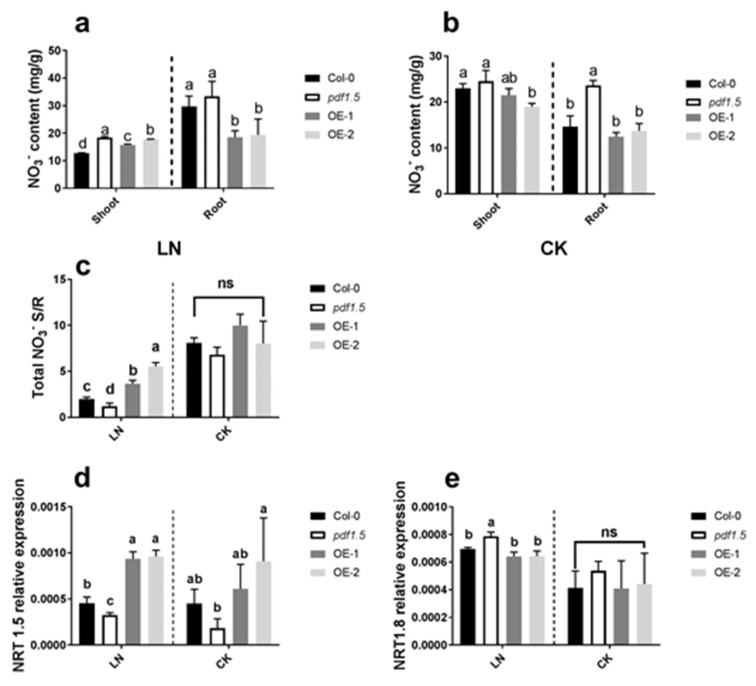
The involvement of *AtPDF1.5* in NO_3_^–^ distribution in low nitrogen (LN) conditions. (**a**) NO_3_^–^ content for different materials grown in LN conditions. (**b**) NO_3_^–^ content for different materials grown in CK conditions. (**c**) Total NO_3_^–^ shoot/root rate for different materials grown in CK or LN conditions. (**d**) *AtNRT1.5* relative expression for different materials grown in CK or LN conditions. (**e**) *AtNRT1.8* relative expression for different materials grown in CK or LN conditions. Bar height represents mean and error bars indicate standard deviation. The statistical analysis was performed on different treatment genotypes. Six replicates were used for measurements.

Our analysis of NO_3_^−^ distribution under LN conditions revealed that, relative to the wild-type Col-0, *pdf1.5*, OE-1, and OE-2 seedlings all had higher shoot NO_3_^−^ contents, whereas OE-1 and OE-2 had lower root NO_3_^−^ contents (Figure 3a). Under CK conditions, the *pdf1.5* mutant had a higher root NO_3_^−^ content than the wild-type Col-0 (Figure 3b). Regarding the shoot/root total NO_3_^−^ ratio, relative to the wild-type Col-0 under LN conditions, the ratio for OE-1 and OE-2 plants was significantly higher, whereas for *pdf1.5,* the ratio was significantly lower under LN conditions (Figure 3c). With regard to *AtNRT1.5* expression, we found that under LN conditions, compared with wild-type Col-0, *pdf1.5* mutant seedlings were characterized by a lower expression, whereas OE-1 and OE-2 plants showed a higher expression (Figure 3d). In contrast, under LN conditions, the *pdf1.5* mutant showed higher *AtNRT1.8* expression than the wild-type Col-0 (Figure 3e).

### 2.3. AtPDF1.5 Enhances Cd Tolerance and Accumulation in A. thaliana

**Figure 4:**
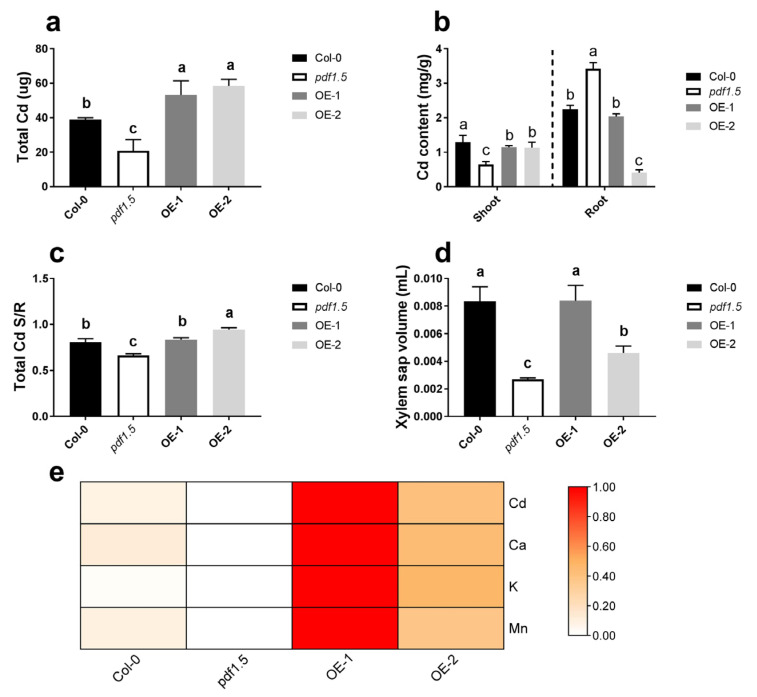
The involvement of *AtPDF1.5* in cadmium (Cd) accumulation and distribution. (**a**) Total Cd for different materials grown in Cd conditions. (**b**) Cd content for different materials grown in Cd conditions. (**c**) Total Cd shoot/root rate for different materials grown in Cd conditions. (**d**) Xylem sap volume for different materials grown in Cd conditions. (**e**) Total Cd, Ca, K and Mn for different materials grown in Cd conditions. Data were normalized with zero-to-one method. Bar height represents mean and error bars indicate standard deviation. Different letters on bars indicate significant differences based on Tukey’s HSD (*p* < 0.05). The statistical analysis was performed on different treatment genotypes. Six replicates were used for measurements.

Based on phenotype, we found that under CK conditions, there were no significant differences in total biomass between either *pdf1.5* or the OE-1 and OE-2 seedlings and those of the wild-type Col-0. In contrast, in seedlings treated with Cd, total biomass was lower in *pdf1.5* and significantly higher in OE-1 and OE-2 (Figure 1a,b). Subsequent studies examining plant tolerance to Cd revealed that, compared with the wild-type Col-0, *pdf1.5* seedlings had a lower total Cd, shoot Cd content, shoot/root total Cd ratio, and xylem sap volume (Figure 4a–d), but a higher root Cd content (Figure 4b). OE-1 and OE-2 had a higher total Cd and shoot Cd content than the wild-type Col-0 (Figure 4a,b). Subsequent analysis of the cationic contents of the xylem sap revealed that *AtPDF1.5* might be involved in Cd allocation and influence Ca, K, and Mn distribution (Figure 4e).

### 2.4. AtPDF1.5 Expression and Subcellular Localization

**Figure 5:**
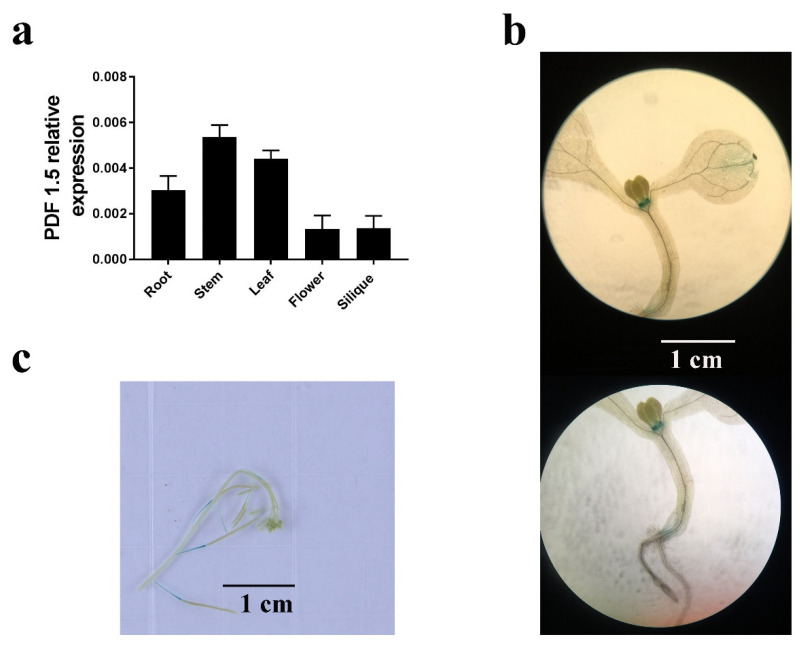
Expression pattern of *AtPDF1.5*. (**a**) Analysis of the relative expression level of *AtPDF1.5* in the different *A. thaliana* tissues by quantitative PCR (qPCR). Tissues were harvested from 4-week-old hydroponically grown plants (roots, stems, leaves, flowers, and siliques). Actin was used as the internal control. (**b**,**c**) Histochemical localization of β-glucuronidase (GUS) activity in transgenic plants expressing the GUS reporter gene under the control of the proAtPDF1.5 promoter. Bar height represents mean and error bars indicate standard deviation. Different letters on bars indicate significant differences based on Tukey’s HSD (*p* < 0.05). The statistical analysis was performed on different treatment genotypes. Six replicates were used for measurements.

**Figure 6:**
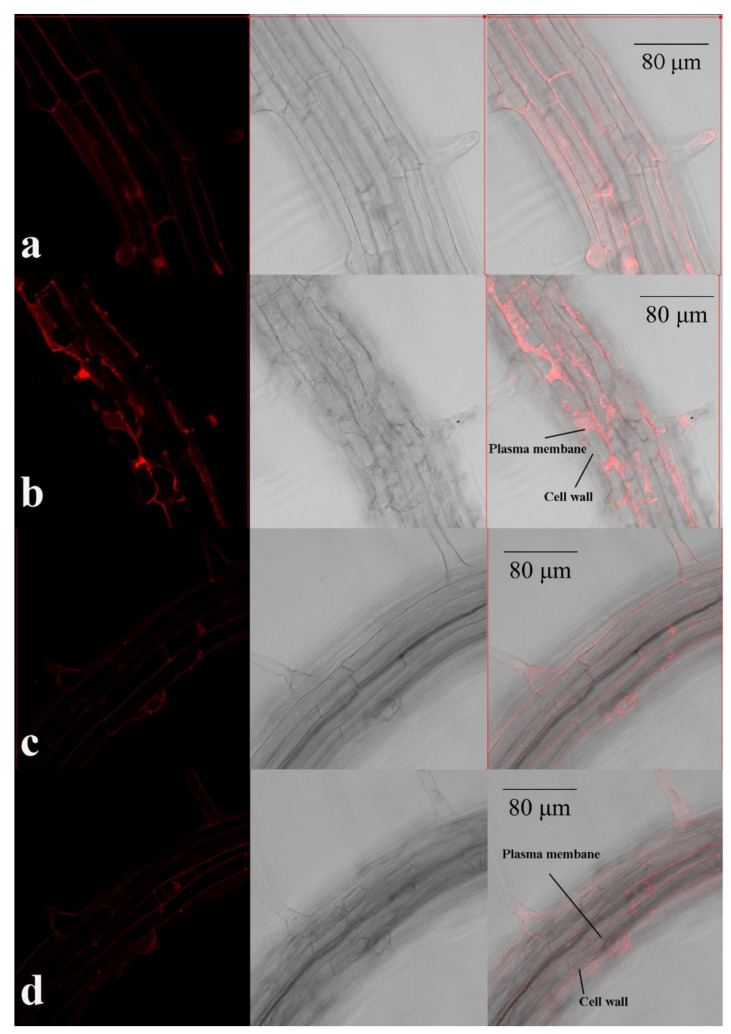
Subcellular localization of *AtPDF1.5* in roots. (**a**) 35S::mRFP before sucrose treatment. (**b**) 35S::mRFP after sucrose treatment. (**c**) 35S::AtPDF1.5-mRFP before sucrose treatment. (**d**) 35S::AtPDF1.5-mRFP after sucrose treatment.

To determine the mechanisms underlying the observed differences in element distribution, we examined the expression and subcellular localization of *AtPDF1.5*. A high expression of *AtPDF1.5* was detected predominantly in the roots, stems, and leaves (Figure 5a). Subsequent GUS expression analysis indicated that *AtPDF1.5* was highly expressed in nodes and the carpopodium (Figure 5b,c). To determine the subcellular localization of *AtPDF1.5*, we used 35S::*AtPDF1.5*-mRFP transgenic plants and 30% sucrose solution to isolate the root cells from the plasma wall. Confocal microscopy revealed that *AtPDF1.5* is located in the cell wall and cytoplasm (Figure 6).

### 2.5. AtPDF1.5 Is Involved in the Allocation of Multiple Metals in A. thaliana

**Figure 7:**
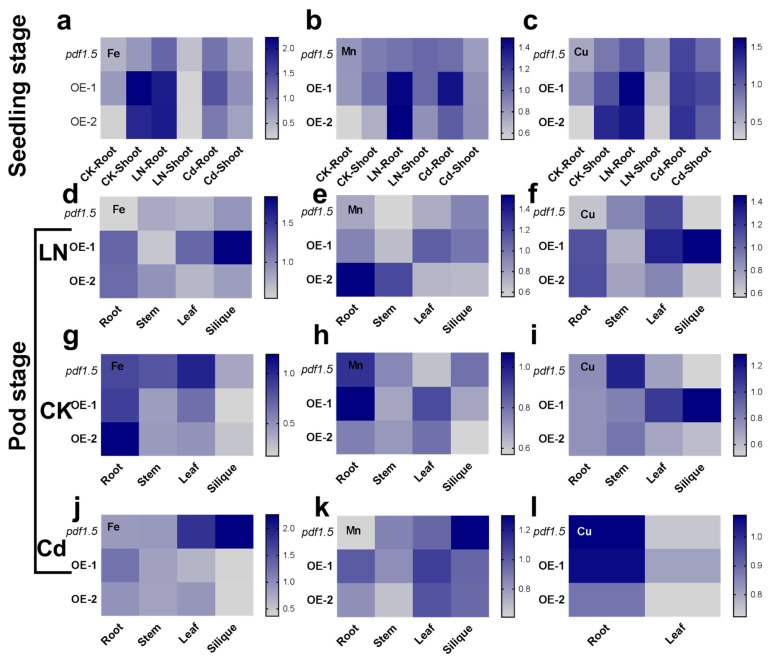
The involvement of *AtPDF1.5* in Fe, Mn, and Cu distributions in the control treatment (CK), low nitrogen (LN), or cadmium (Cd) conditions. (**a**,**d**,**g**,**j**) Fe content for seedling and pod stage in CK or LN or Cd conditions. (**b**,**e**,**h**,**k**) Mn content for seedling and pod stage in CK or LN or Cd conditions. (**c**,**f**,**i**,**l**) Cu content for seedling and pod stage in CK or LN or Cd conditions. Different cationic content was divided by Col-0 relatively. Six replicates were used for measurements.

We analyzed the levels of different cations under CK, LN, and Cd conditions at both the seedling and pod stages. We used different plant cationic contents divided by Col-0 and used these values to generate heat maps (Figure 7). At the seedling stage, relative to the wild-type Col-0 under the CK condition, *AtPDF1.5* contributed to a higher distribution of Fe in shoots (Figure 7a), whereas under LN conditions, *AtPDF1.5* contributed to a higher accumulation of Fe, Mn, and Cu in roots (Figure 7a–c). Under Cd conditions, *AtPDF1.5* was associated with higher Mn and Cu accumulations in roots (Figure 7b,c).

At the pod stage, relative to the wild-type Col-0 under CK conditions, *AtPDF1.5* contributed to higher Fe levels in roots (Figure 7g), higher Mn levels in leaves (Figure 7h), and lower Cu levels in stems (Figure 7i). Under LN conditions, *AtPDF1.5* contributed to higher Fe, Mn, and Cu levels in roots (Figure 7d–f). In contrast, under Cd conditions, *AtPDF1.5* contributed to lower Fe and Mn levels in siliques (Figure 7j,k), but higher Mn levels in leaves (Figure 7k).

**Figure 8:**
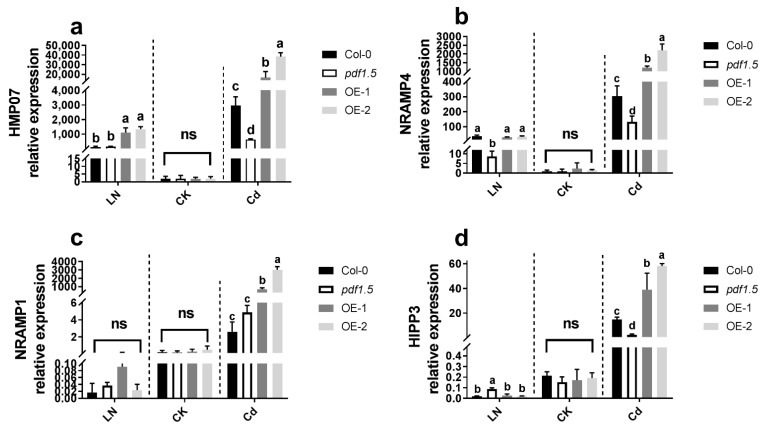
*AtPDF1.5* involved the gene’s expression for metal transporter. (**a**) *AtHMP07* relative expression for different materials grown in LN or CK or Cd conditions. (**b**) *AtNRAMP4* relative expression for different materials grown in LN or CK or Cd conditions. (**c**) *AtNRAMP1* relative expression for different materials grown in LN or CK or Cd conditions. (**d**) *AtHIPP3* relative expression for different materials grown in LN or CK or Cd conditions. Bar height represents mean and error bars indicate standard deviation. Different letters on bars indicate significant differences based on Tukey’s HSD (*p* < 0.05). The statistical analysis was performed on different treatment genotypes. Six replicates were used for measurements.

To determine the mechanisms underlying the observed differences in metal distribution, we examined the expression of selected metal transporter genes (*AtHMP07*, *AtNRAMP4*, *AtNRAMP1,* and *AtHIPP3*) in *Arabidopsis* seedlings subjected to the CK, LN, and Cd treatments (Figure 8). We found that, relative to the wild-type Col-0, there were no significant differences in the expression of these genes in *pdf1.5*, OE-1, or OE-2 seedlings, whereas under LN conditions, the expression of *AtHMP07* was significantly higher in OE-1 and OE-2 seedlings, *AtNRAMP4* was expressed in *pdf1.5*, and a high expression of *AtHIPP3* was detected in *pdf1.5*. Under Cd conditions, compared with the wild-type Col-0, *AtHMP07*, *AtNRAMP4*, *AtNRAMP1*, and *AtHIPP3* were highly expressed in OE-1 and OE-2 seedlings, whereas *AtHMP07*, *AtNRAMP4,* and *AtHIPP3* expression levels were downregulated in the *pdf1.5* mutant (Figure 8).

## 3. Discussion

Previous studies on AtPDF1 have focused primarily on their roles in Zn tolerance and disease resistance [33]. For example, AtPDF1.2 has been shown to be induced by the coordinated interaction of the jasmonic acid and ethylene signaling pathways, which may function to induce defense-related gene expression in *A. thaliana* [42,43]. However, there is currently relatively limited information available regarding the role of AtPDF1 genes in LN tolerance and Cd resistance [37,44], particularly concerning the similarity of the underlying mechanisms.

In the present study, based on the analyses of the *pdf1.5* mutant line and loss of function material (*pdf1.5-1* and *pdf1.5-2*), two *AtPDF1.5* overexpression lines (OE-1 and OE-2), and the wild-type Col-0 (Figure 1), we found that the expression of *AtPDF1.5* was upregulated in response to both the LN and Cd treatments, particularly the latter, in which there was a 61-fold increase in root expression (Figure S1).

### 3.1. AtPDF1.5 Enhances Adaptation to LN by Regulating AtNRT1.5 and AtNRT1.8 Expression

Considering that the major function of *AtPDF1.5* appears to be nutrient element distribution, and compared with CK, the root system architecture was significantly changed in LN or Cd conditions, but the shoot/root ratio was not significantly different (Figure S3). Therefore, we did not examine nitrogen uptake further (Figure 5). *AtPDF1.5* is a small peptide, and it has been previously reported that small peptides can affect transporters through their downstream receptors and long-distance signals by binding to receptors [45,46,47]. Therefore, we hypothesized that *AtPDF1.5* might regulate transporters’ expression through these two ways.

With respect to nitrogen distribution [48], we found that *AtPDF1.5* is involved in NO_3_^−^ distribution under LN conditions but not under CK conditions (Figure 3a–c). To determine the mechanisms underlying this activity, we examined the expression of *AtNRT1.5* and *AtNRT1.8* under both LN and CK conditions [48,49]. Concerning *AtNRT1.5* in response to LN [46], we observed that, relative to the wild-type Col-0, *AtNRT1.5* was upregulated in the overexpression lines OE-1 and OE-2 and downregulated in the *pdf1.5* mutant (*pdf1.5-1*), whereas under CK conditions, the expression of *AtNRT1.5* in *pdf1.5* seedlings did not differ significantly from that in wild-type Col-0 [8]. Contrastingly, we found that under LN conditions, the expression of *AtNRT1.8* in *pdf1.5* was upregulated, relative to that in the wild-type Col-0 and the two overexpression lines [9,49]. However, no significant differences were detected under CK conditions. These observations indicate that *AtPDF1.5* may enhance the acclimation of *Arabidopsis* to LN by involving the expression of *AtNRT1.5* and *AtNRT1.8* (Figure 3d,e).

### 3.2. AtPDF1.5 Enhances Cd Tolerance by Increasing Cd Transport to Shoots

We found that under the CK treatment, there were no significant differences among the wild-type Col-0, *pdf1.5*, OE-1, and OE-2 seedlings with respect to phenotype (Figure 1a,b). However, in the vegetative growth period when exposed to Cd, the mutant seedlings had a lower total biomass than the wild-type Col-0 seedlings, whereas the overexpression lines OE-1 and OE-2 had a higher total biomass (Figure 1a,b). Furthermore, relative to the wild-type Col-0 seedlings, the *pdf1.5* mutant had a lower Cd accumulation, whereas OE-1 and OE-2 had a higher Cd accumulation (Figure 4a). These observations are consistent with the findings previously reported for *AtPDF2.5* [39]. We also obtained lower values for the Cd content, shoot/root total Cd ratio, xylem sap volume, and metal content in xylem sap in the *pdf1.5* mutant (Figure 4b–e). Collectively, these observations indicate that *AtPDF1.5* may enhance Cd transport to shoots, as indicated by the respective Cd contents of the shoots and roots (Figure 4b). We suspect *AtPDF1.5* could promote the chelation efficient of Cd, and the low shoot/root total Cd ratio may protect the nutrient absorption of roots more effectively. Our results showed that the transport of Cd was not enhanced; meanwhile, the overexpression resulted in an increase in total Cd and a decrease in aboveground content due to the increase in biomass. *PDF1.5* is localized to the cell wall and may play a role in detoxifying cadmium by chelating cadmium to the cell wall. Consequently, our findings indicate that *AtPDF1.5* enhances Cd tolerance by regulating the transport of this metal [11,50,51].

### 3.3. AtPDF1.5 Enhances Cd Transport and Affects the Transport of Other Cations

Our analysis of the Cd, K, Ca, and Mn levels in xylem sap indicates that *AtPDF1.5* may mediate the distribution of Cd and influence the transport of other cations (Figure 4e and Figure 7) [52]. Therefore, we examined the levels of cations in various tissues and at different growth stages [53,54]. We found that *AtPDF1.5* is involved in Cd allocation and plays a role in determining the distribution of other cations (Figure 7).

To assess the associated regulatory mechanisms, we examined the expression of selected metal transport genes [28,29,55]. Under CK conditions, we detected no significant differences in the expression of *AtHMP07*, *AtNRAMP4*, *AtNRAMP1*, and *AtHIPP3* in the *pdf1.5* mutant and overexpression lines and the wild-type Col-0. However, in response to LN treatment, *AtHMP07* was upregulated in the OE-1 and OE-2 overexpression lines (Figure 8a), *AtNRAMP4* was downregulated in the *pdf1.5* mutant (Figure 8b) [25,26,27], and *AtHIPP3* was upregulated in the *pdf1.5* mutant (Figure 8d) [31]. Moreover, in seedlings treated with Cd, relative to the wild-type Col-0, the expression of *AtHMP07*, *AtNRAMP4*, and *AtHIPP3* was downregulated in the *pdf1.5* mutant and upregulated in the overexpression lines (Figure 8a,b,d), and *AtNRAMP1* was also characterized by the upregulated expression of overexpression lines (Figure 8c).

Based on these observations, we speculate that *AtPDF1.5* enhances plant adaptation to LN conditions and the presence of Cd by modifying the distribution of nutrient elements [56,57]. As a small molecular protein [44,58,59,60], we speculate that *AtPDF1.5* may be involved in the processing or transmission of signal substances and play an important role in the remediation of Cd pollution and LN acclimation. As a key member of the *AtPDF1* family [32,61], *AtPDF1.5* warrants further research, as it may have other essential functions in environmental acclimation. We have studied the root expression of *AtPDF1.5* in *Brassica napus* under CK and LN conditions and obtained the same results (Figure S4). There is a need for the time-dependent application of omics technologies to compare various *pdf1.5* mutants and dissect the order of events.

## 4. Materials and Methods

### 4.1. Materials and Growth Conditions

Seeds of wild-type *A. thaliana* ecotype Col-0 were obtained from the Shanghai Institute of Plant Physiology and Ecology. *AtPDF1.5* (AT1G55010) knockout mutant *pdf1.5-1* (SALK_151733) and loss of function material *pdf1.5-2* (SALK_070545) were acquired from the Eurasian *A. thaliana* Stock Centre (uNASC; http://arabidopsis.info/, 29 December 2019). After validation, we renamed knockout mutant *pdf1.5-1* (SALK_151733) to “*pdf1.5*” for the next study.

We generated the *AtPDF1.5* overexpression lines OE-1 and OE-2. The seeds of Col-0, *pdf1.5* mutant, loss of function material, and *AtPDF1.5* overexpression lines were germinated and grown in a greenhouse (300 μmol photons m^−2^ s^−1^, 16 h photoperiod, 22 °C) for 10 d. At the two-leaf growth stage, each seedling was transferred to a 5-L pot, and the pots were given different treatments. For the CK treatment (control conditions), the seedlings were provided with a nutrient solution containing 1.25 mM KNO_3_, 0.625 mM KH_2_PO_4_, 1.25 μM Fe-EDTA, 0.5 mM MgSO_4_, 0.5 mM Ca(NO_3_)_2_, 0.05 μM NaMoO_4_, 0.125 μM CuSO_4_, 0.25 μM ZnSO_4_, 3.5 μM MnCl_2_, and 17.5 μM H_3_BO_3_. For the LN treatment, plants were provided with a nutrient solution containing 0.15 mM KNO_3_ and 0 mM Ca(NO_3_)_2_, and K and Ca were supplied as KCl and CaCl_2_, respectively [48]. Other components were the same as those used in the CK treatment. For the Cd (10 μM CdCl_2_) treatment, all plants were grown in the CK nutrient solution for the initial 14 d and thereafter grown for a further 7 d with 10 μM CdCl_2_ supplementation. The pH of the medium was adjusted to 5.8, and the medium was renewed at 4 d intervals [37]. Six replicates were used for all measurements.

### 4.2. Preparation of DNA Constructs and Plant Transformation

We initially used polymerase chain reaction (PCR) to amplify a 580 bp genomic fragment immediately upstream of the *AtPDF1.5* start codon, using the *ProAtPDF1.5* primer pair F: CGACGGCCAGTGCCAAGCTTGTGTGATTAATGTTATGTGT and R: GACTGACCACCCGGGGATCCATGACTTACTACTTAGATTT. The amplified promoter fragment was then sub-cloned into a pCAMBIA1300-GUS binary vector [39]. Constructs were transferred into *A. thaliana* using the floral dip method [62]. Transgenic plants were selected using hygromycin B and confirmed by sequencing. To determine the subcellular localization of *AtPDF1.5* in *A. thaliana*, the 35S::mRFP fragment was recovered from 35S::mRFP/PA7 via *Hin*dIII/*Sac*I restriction digestion and the resulting 35S::mRFP fragment was inserted into pCAMBIA1300 to generate the 35S::mRFP/pCAMBIA1300 construct. The coding sequence of *AtPDF1.5* was PCR amplified using the *AtPDF1.5* primer pair F: CGGGGGACTCTAGAGGATCCTGGCTAAGTTTTGTACCACC and R: TCGGAGGAGGCCATACTAGTACCAGCGCAATATCCATCAT, and the amplified *AtPDF1.5* coding sequence fragment was sub-cloned into the binary vector 35S::mRFP/pCAMBIA1300 to generate the construct *35S*::*AtPDF1.5* -mRFP/pCAMBIA1300, as described by Luo et al. [39,44]. Six replicates were used for measurements.

### 4.3. Expression, β-Glucuronidase (GUS) Histochemical Analyses, and Subcellular Localization

Total RNA was extracted from the collected tissues (root, stem leaf, flower, silique, and seedling) using TRIZOL reagent according to the manufacturer’s instructions (Invitrogen, Carlsbad, CA, USA). Complementary DNAs were synthesized using a PrimeScript™ RT Kit with gDNA Eraser (Perfect Real Time; TAKARA, Shanghai, China) following the manufacturer’s protocol. The relative expression levels of target genes were determined by reverse transcription quantitative PCR (RT-qPCR) performed using an Applied Biosystems StepOne™ Real-Time PCR System with SYBR Premix Ex-Taq (TAKARA), according to the manufacturer’s instructions. The relative expression of detected genes was normalized to that of the reference gene using the delta–delta Ct (threshold cycle, ΔΔCt) method. The primers used in this study are listed in Table S1, and the expression level of *AtPDF1.5* was normalized to that of *Actin.* Histochemical staining, driven by the *ProAtPDF1.5* promoter, was performed using a GUS histochemical analysis kit (Real-Times Biotechnology Co., Ltd., Beijing, China). GUS staining patterns in root tissues were observed under an Olympus BX51 microscope and photographed using a Fujifilm X-A3 camera [39]. Six replicates were used for measurements.

To determine the subcellular localization of *AtPDF1.5* in *A. thaliana*, the coding sequence of *AtPDF1.5* was amplified by PCR using primers AtPDF1.5F and AtPDF1.5R (Supplementary Table S1) and then subcloned to generate the construct 35S::mRFP/1300. The resulting fragments were fused in-frame to the 5′ terminus of the monomer red fluorescent protein (mRFP) gene to generate the 35S::AtPDF1.5-mRFP/pCAMBIA1300 constructs. These constructs were modified by replacing the 35S promoter with the native promoter proAtPDF1.5, resulting in the proAtPDF1.5::AtPDF1.5-mRFP/pCAMBIA1300 constructs, which were transformed into *A. thaliana* using the floral dip method [37]. Root tissues of the resulting transgenic lines were first subjected to mRFP imaging using confocal microscopy (LSM880; Zeiss) and then reimaged after being treated with 30% sucrose.

### 4.4. Nitrogen Concentration Assay

The dry biomass and nitrogen concentration of treated plants were determined at the seedling stage (21 d after transplantation). Plants were harvested and oven-dried to constant weight by heating at 105 °C for 30 min, followed by a slow drying at 65 °C. Nitrogen concentrations were determined using the Kjeldahl method [63]. The entire tissues of dried plants were initially digested with H_2_SO_4_ and then subjected to analysis using a Foss Auto Analyzer Unit (AutoAnalyzer 3; SEAL Analytical, Inc., Nordsted, Germany). Six replicates were used for measurements. Physiological nitrogen-use efficiency was calculated using the following equation [40]:

Physiological nitrogen-use efficiency = Plant total biomass/Plant total nitrogen.

### 4.5. Xylem Sap Collection and NO_3_^−^ Concentration Assay

We used the method of Wu to collect xylem sap [40]. Using 21-day-old plants, stems were cut 1 cm above the intact roots, which were left to grow in the respective nutrition solutions. Xylem saps were collected by 10 μL pipettors and saved in pre-weighed 2 mL ep tubes. Tubes were placed on ice when the experiment started. After 45 min, the volume of xylem collected was calculated as the weight gain of the ep tube. NO_3_^−^ concentrations were determined using the aforementioned Foss Auto Analyzer Unit. Six replicates were used for measurements.

### 4.6. Cation Concentration Assay

Analysis of cation concentration was performed as described by Gong and Luo [39,64]. Dried samples of whole plants and xylem sap were initially digested with 70% HNO_3_. The samples were washed with distilled water twice before drying. Cation concentrations in the resulting digests were determined using inductively coupled plasma mass spectrometry (ICP-MS: ELAN DRC-e; PerkinElmer, Norwalk, CT, USA). Six replicates were used for measurements.

### 4.7. Statistical Analysis

Statistical analyses were performed using SPSS software (version 25.0; SPSS Statistics Inc., Chicago, IL, USA). All experiments were conducted using a completely randomized design. Data were compared by two-way ANOVA and Tukey’s HSD post hoc test. Differences were considered significant at the *p* < 0.05 level [65].

## 5. Conclusions

In the long-term process of breeding, people often focus on the breeding of high-yield varieties but ignore their resistance to environmental stress. Cultivars with good growth conditions have poor resistance to adversity, while cultivars with strong resistance to adversity have poor growth conditions. Deep understanding of the relationship between plant growth and stress resistance is of great significance for improving crop yield and resistance. Our study revealed the function of *AtPDF1.5*. As a short peptide of a small molecule, we suspect that AtPDF1.5 may play a key role in chelation and signal transmission; the mechanism for cadmium tolerance may be chelation, and for LN it may be signaling function, thus improving the adaptation for LN and Cd stress in plants. Our study provided a new insight for breeding high-yield and high-resistance crops, especially in phytoremediation for toxic metal-polluted areas. Based on our research, it is not difficult to explore the nutritional element-rich product in crop production.

## Supplementary Materials

The following are available online at https://www.mdpi.com/article/10.3390/ijms221910455/s1, Figure S1: The *AtPDF1.5* expression of Col-0 was induced by low nitrogen (LN) and cadmium (Cd). Figure S2: Materials verification for *At**PDF1.5*. Figure S3: The root phenotype for materials grown in low nitrogen (LN) or cadmium (Cd) condition. Figure S4: *BnPDF1.5* relative expression of root for two contrasting *Brassica napus* genotypes grown in the control treatment (CK) or low nitrogen (LN) condition. Table S1: Primers used in this study.

## Abbreviations

CK: Control; GUS: β-glucuronidase; HIPP3: Heavy metal-associated isoprenylated plant protein-3; LN: Low nitrogen; NRAMP1: Natural resistance-associated macrophage protein-1; NRAMP4: Natural resistance-associated macrophage protein-4; NRT1.5: Nitrate transporter 1.5; NRT1.8: Nitrate transporter 1.8; PDF: Plant defensin; Cd: Cadmium.
