# Supplementary material for: PDF1.5 Enhances Adaptation to Low Nitrogen Levels and Cadmium Stress
Source: Int J Mol Sci. 2021 Sep 28;22(19):10455. doi: 10.3390/ijms221910455 (PMC8509053; doi:10.3390/ijms221910455)
Supplement: Supplementary file 1 [file ijms-22-10455-s001.zip › ijms-1272071-supplementary.pdf]

Supplementary Date

Table S1 Primers used in this study.

Name	Forward Sequence	Reverse Sequence
	AtPDF1.5 transgenic plant construct (*Arabidopsis thaliana)*	
PAtPDF1.5	CGACGGCCAGTGCCAAGCTTGTGTGATTAATGTTATGTGT	GACTGACCACCCGGGGATCCATGACTTACTACTTAGATTT
AtPDF1.5	CGGGGGACTCTAGAGGATCCTGGCTAAGTTTTGTACCACC	TCGGAGGAGGCCATACTAGTACCAGCGCAATATCCATCAT
	PDF1.5 mutant verification	
pdf1.5-1	TGATTCCAGGCTCAAAACATC	TGATCTGTACCATGGGTAGCC
LBb1.3	ATTTTGCCGATTTCGGAAC	
	Quantitative RT-PCR (*Arabidopsis thaliana)*	
Real-AtPDF1.5	TTGGTTGCTCTTGTTCTCT	ATCTCTGCACTGATAATCGT
AtNRT1.5	ATCGCTATCGCTATCATCAT	TCCGTCATACATCTCCTCT
AtNRT1.8	CTCAGTGTCGCCTTCTTC	CATCGCCATTATCGCAATC
AtHMP07	GAGTCCACGATGTTCAGATA	GCCGTTCTTGATAGTATCCA
AtNRAMP4	CGAAGAAGAAGACGCTGAT	CAATCTCCGCCATAATCCA
AtNRAMP1	GTAGTTGCTTGTGACATACC	CCGAGTGGAGGAAGAGAT
AtHIPP3	CCATTGTCAAGGTTGTATCG	CAAGCATTCGGATTCTCATC
Actin	GGTCGGGACCTCACTGA	CAACGGAATCTCTCAGCTCC

	TTC	
	Quantitative RT-PCR (*Brassica napus*)	
BnaEF1-α	GCCTGGTATGGTTGTGACC T	GAAGTTAGCAGCACCCTTGG
BnPDF1.5	CATCTTCGCTGCTCTCATT	CAAGAACCATCTCGTGCTT

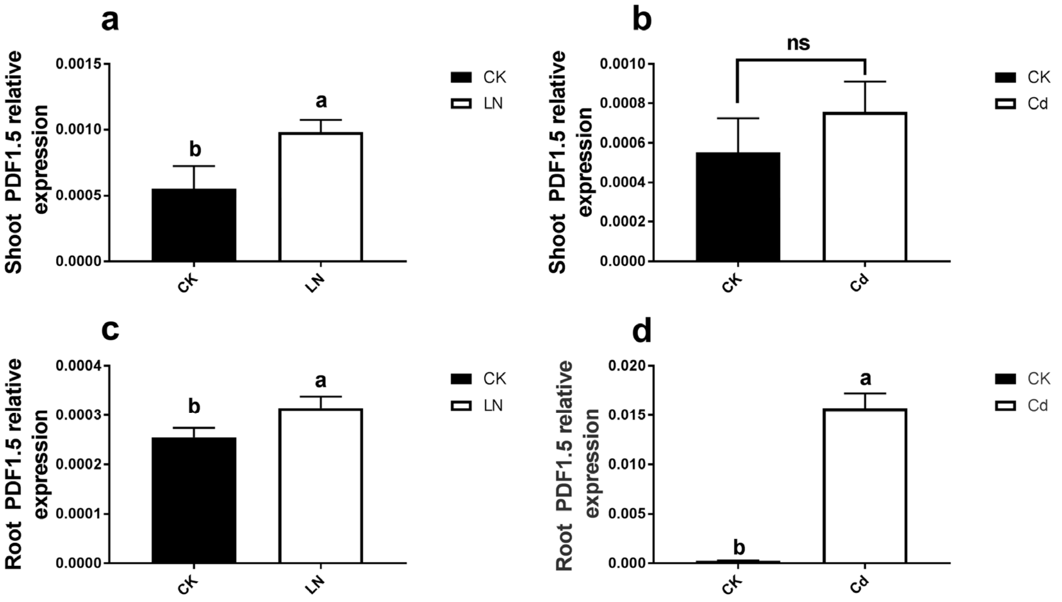

Figure S1. The *AtPDF1.5* expression of Col-0 was induced by low nitrogen (LN) and cadmium (Cd).

(a) Shoot *AtPDF1.5* relative expression in LN and CK conditions. (b) Shoot *AtPDF1.5* relative expression in Cd and CK conditions. (c) Root *AtPDF1.5* relative expression in LN and CK conditions. (d) Root *AtPDF1.5* relative expression in Cd and CK conditions. Bar height represents mean and error bars indicate standard deviation. Different letters on bars indicate significant differences based on student's t test ($p < 0.05$). The statistical analysis was performed on different treatments genotypes. Six replicates were used for measurements.

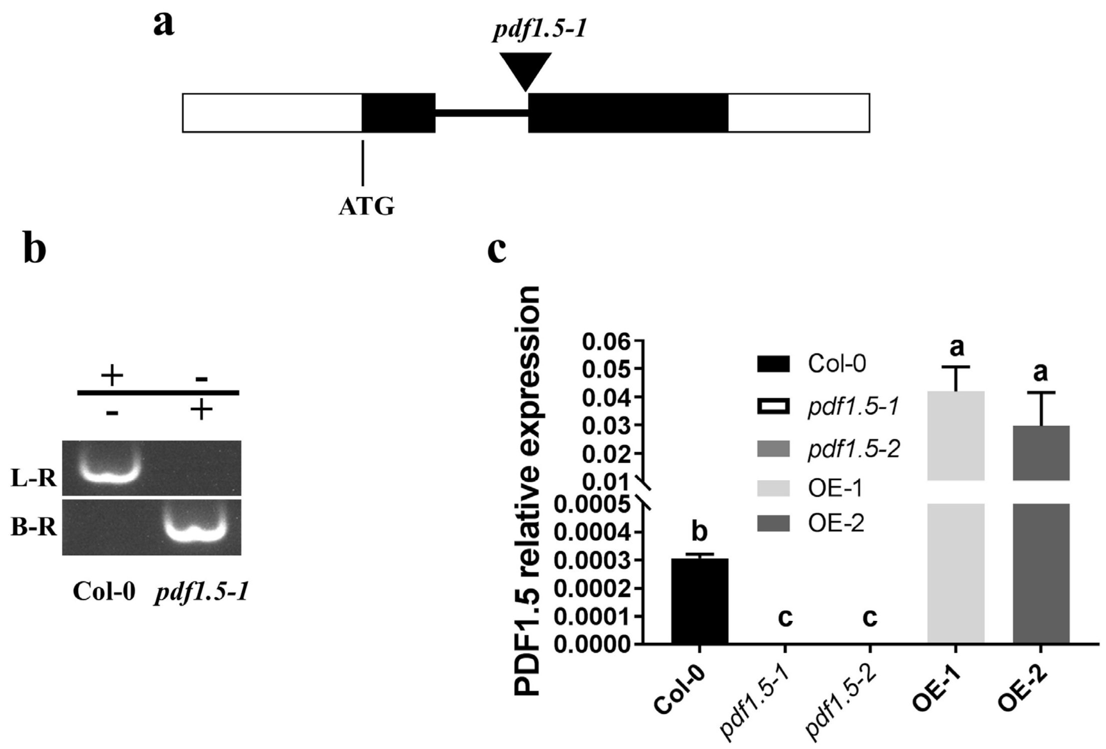

Figure S2. Materials verification for *AtPDF1.5*.

(a) Schematic representation of the allelic transfer DNA insertion line *pdf1.5–1* (SALK_151733). The white blocks represent 5′−3′ UTR, and the line connected to the two black blocks represents intron. (b) *pdf1.5–1* (SALK_ 151733) homozygous validation, we used DL2000 as the marker. (c) Analysis of the relative expression of *AtPDF1.5* in different *Arabidopsis thaliana* root tissues, using quantitative PCR. Root tissues were harvested from 3-week-old hydroponically-grown plants. Actin was used as the internal control. Root tissues were harvested either from 3-week-old hydroponically-grown plants. Actin was used as the internal control. Bar height represents the mean, and error bars indicate standard deviation. Different letters on bars indicate significant differences based on Tukey’s HSD ($p < 0.05$). The statistical analysis was performed on different treatment genotypes. Six replicates were used for measurements.

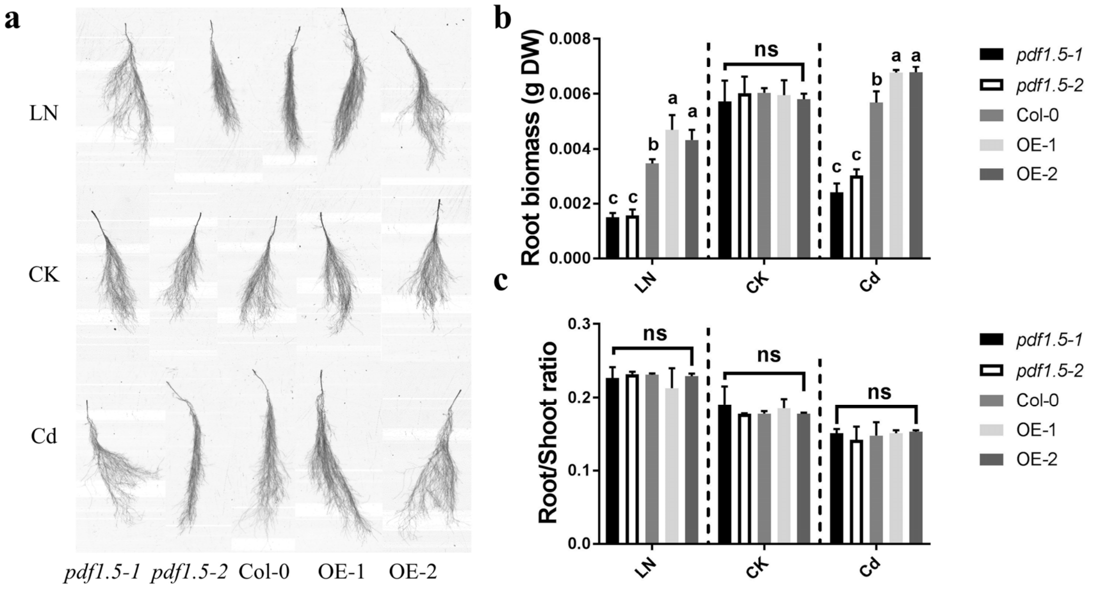

Figure S3. The root phenotype for materials grown in low nitrogen (LN) or cadmium (Cd) condition.

(a) Root phenotype for different materials, materials were harvested either from 3-week-old hydroponically-grown plants in LN or Cd or CK condition.

(b) Root biomass for different materials. (c) Root / shoot ratio for different materials. Bar height represents mean and error bars indicate standard deviation. Different letters on bars indicate significant differences based on Tukey's HSD ($p < 0.05$). The statistical analysis was performed on different treatments genotypes. Six replicates were used for measurements.

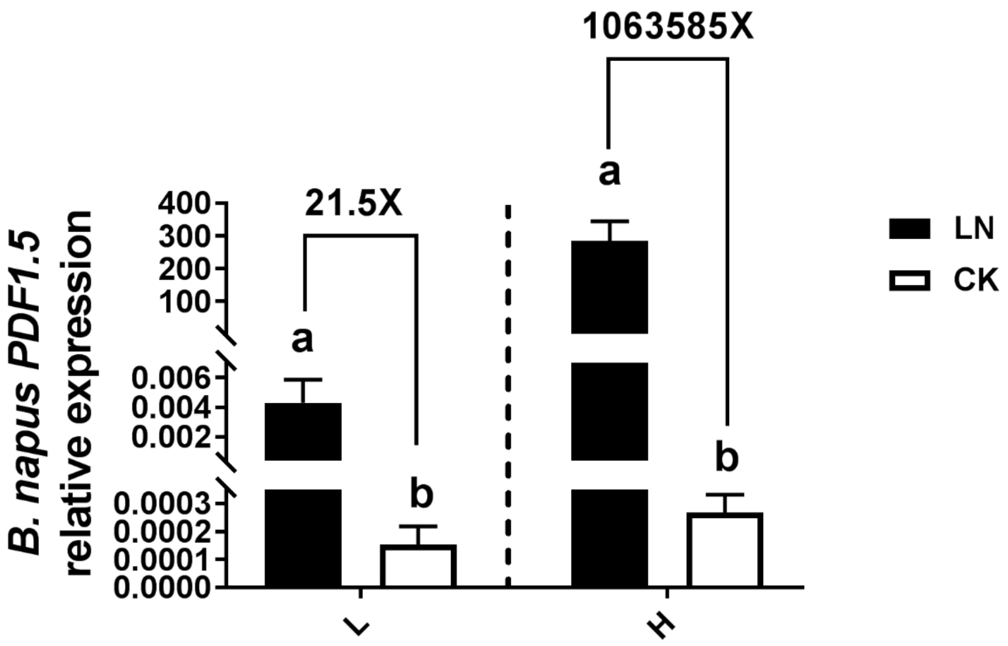

Figure S4. *BnPDF1.5* relative expression of root for two contrasting *Brassica napus* genotypes grown in the control treatment (CK) or low nitrogen (LN) condition.

B. napus seedlings (L: 814, H: xiangyou 15) were grown in ceramic pots (22 cm × 15 cm × 7 cm) fixed to the perforation of a PVC plank by a sponge, and containing different nutrition solution (CK: 15 mM NO_3^--N or LN: 1 mM NO_3^--N) 20 days. The nutrient solution was replaced every 4 days (Wu et al., 2019). *BnaEF1-α* was used as the internal control. Bar height represents mean and error bars indicate standard deviation. Different letters on bars indicate significant differences based on Tukey's HSD ($p < 0.05$). The statistical analysis was performed on different treatments genotypes. Six replicates were used for measurements.
